# Examination of performance of the Center for Epidemiologic Studies Depression Scale Short Form 10 among African youth in poor, rural households

**DOI:** 10.1186/s12888-018-1774-z

**Published:** 2018-06-18

**Authors:** Kelly Kilburn, Leah Prencipe, Lisa Hjelm, Amber Peterman, Sudhanshu Handa, Tia Palermo

**Affiliations:** 1 Institute for Global Health and Infectious Diseases, University of North Carolina, Bioinformatics CB# 7030, Chapel Hill, NC 27599-7030 USA; 2 UNICEF Office of Research – Innocenti, Piazza SS. Annunziata, 12, 50122 Florence, Italy; 3 UNICEF East and Southern Africa Regional Office, P.O. Box 44145, Nairobi, 00100 Kenya; 4 Department of Public Policy, University of North Carolina, Abernethy Hall CB #3435, Chapel Hill, NC 27599-3435 USA

**Keywords:** Mental health, Depression, Youth, Measurement, Africa, Cash transfers

## Abstract

**Background:**

Youth mental health has emerged as a pressing global issue. However, to advance research gaps in low-income settings, we need valid measures of common mental health disorders. Using primary data collected in five countries (Kenya, Malawi, Tanzania, Zambia, and Zimbabwe), this study aims to assess the psychometric properties of the commonly used 10-item Center for Epidemiological Studies Depression (CES-D 10) scale among poor, disadvantaged youth populations in sub-Saharan African (SSA).

**Methods:**

Youth samples from each country (sample sizes ranging from 651 to 2098) come from large household surveys with youth modules, collected for impact evaluations of cash transfer programs targeted to poor families. For each sample, we assessed internal consistency (alpha), conducted factor analysis, and then examined construct validity and measurement invariance. We performed both exploratory (EFA) and confirmatory factor analysis (CFA) to examine and confirm the structure of the CES-D 10 for each country and then used multigroup CFA to assess measurement invariance across gender and age. Multivariate analyses were conducted to assess construct validity via test of the relationship between CES-D 10 and background characteristics.

**Results:**

Results show the CES-D 10 had strong psychometric properties and was a reliable measure of depressive symptoms among disadvantaged youth in SSA. Across countries, there was high internal consistency (Cronbach alphas = 0.70–0.76) and the traditional two-factor solution showed good model fit. Full measurement invariance of the CES-D 10 was supported across gender. Consistent with previous literature on risk factors for depressive symptoms, the CES-D 10 was associated with increasing age, and female gender and being out of school in some locations.

**Conclusions:**

Results from this study support broad use of the CES-D 10 among poor youth populations in SSA. Between one-third and two-thirds of our samples demonstrated depressive symptoms as classified by recommended cut-offs for the CES-D 10, indicating a high burden of mental illness in disadvantaged youth populations. This tool can be used in future efforts to study prevalence and dynamics of depressive symptoms in this population, as well as effectiveness of policies and interventions to improve the mental health of youth in SSA.

**Electronic supplementary material:**

The online version of this article (10.1186/s12888-018-1774-z) contains supplementary material, which is available to authorized users.

## Background

Youth mental health has emerged as a pressing issue globally, but has been largely underacknowledged and under investigated in policy and research to date [1]. Mental illness is a leading cause of death among adolescents and its contribution to the global burden of disease is highest in low- and middle-income countries (LMICs), where the majority of young people live [2]. The importance of attending to youth mental health is compounded by the fact that the onset of mental health issues often occurs during adolescence [3]. This early onset can result in negative and lasting impacts as adolescence is a crucial time period in the developmental process and lays the foundation for health trajectories into adulthood [1]. Mental disorders in adolescence are associated with poor physical, reproductive, and sexual health, in addition to lower educational attainments and risky behaviors such as substance abuse [4]. Given these long-term impacts, action is needed to address knowledge gaps around the mental health needs of vulnerable young people, as well as how policies and programs can benefit this population.

To do this work, however, we need valid measures of common mental health disorders, such as depressive symptoms, to accurately assess rates of mental illness and respond to needs of vulnerable youth populations. Evidence generation on this topic is limited in sub-Saharan African (SSA), a region characterized by high poverty, due to the capability of existing measurement tools [5, 6]. Most instruments for the assessment of mental illness were developed for western populations, and the reliability of these tools in SSA contexts is uncertain because symptoms of mental illness can express differently across cultures [5, 7]. There have been some efforts to validate these tools in SSA among adult populations, however evidence on the reliability and validity of these tools among African youth is scarce [6]. In order to better understand the prevalence and risk factors associated with depressive symptoms in this population, measurement tools must be validated.

Increasingly, the Center for Epidemiological Studies Depression (CES-D) scale is being used in SSA to measure depressive symptoms among youth (see Additional file 1: Table S1), however the evidence on its validity and reliability in these populations has not been assessed among rural, poor populations. The majority of studies examining the psychometric properties and performance of the CES-D in SSA have been conducted among adult populations or higher education students (including university and secondary school students), who represent the most educated and highest socio-economic strata [8–12]. Despite the lack of rigorous validation or examination of the performance of the CES-D in these populations, the scale has been increasingly used, primarily with the objective of correlating depressive symptoms with background and other risk factors. These studies, for example, have sought to analyze the prevalence of depressive symptoms in youth in Rwanda participating in a mentorship program [13, 14], the impact of a cash transfer on depressive symptoms among youth in eligible poor households in Kenya [15], and to assess correlates of depression among youth in Eastern Cape, South Africa participating in an Human Immunodeficicy Virus (HIV) intervention [16]. As such, this study fills a gap by advancing understanding of the performance and psychometric properties of the CES-D among youth in the poorest socio-economic strata residing in rural areas in SSA.

Using primary data from household samples collected in five countries (Kenya, Malawi, Tanzania, Zambia, and Zimbabwe), this study aims to examine the psychometric properties of the 10-item, short-form of the CES-D (CES-D 10) in rural, SSA youth populations. Because we use data from youth in households targeted for cash transfer programs and these programs are for the most poor and vulnerable households, youth in our samples represent those living in most extreme poverty and vulnerability in the region. We also contribute to understanding of the broader literature of measurement of depressive symptoms among youth in SSA and provide translated tools for researchers wishing to utilize these measures in the future.

### CES-D validity and reliability

The CES-D was originally developed in 1976 to measure depressive symptoms in the general adult population (18 and over) in the United States (US) [17], and has also been validated among US adolescents and young adults [18]. Since then, the scale has been widely used and validated as a tool to measure depression among many general and clinical populations across the world. The initial validation of the CES-D scale in the general population showed high internal consistency (Cronbach α = 0.85) [17]. Additional studies have also found that the CES-D has high internal consistency (Cronbach’s alpha scores consistently higher than 0.8) across youth populations in the west [19–21] and among non-western populations [22, 23], including in SSA [8, 24, 25].

In this study, we use the CES-D 10, modified from the original 20-item CES-D questionnaire [26]. The items chosen for the CES-D 10 were those that displayed high correlation with the full 20-item CES-D, but not with each other, to limit redundancy. The CES-D 10 was first validated in a sample of healthy older adults in the US [26], but has since been validated more widely including among elderly Chinese [27], adolescents in France [28] and Canada [29], and among HIV-positive people in Canada [30]. In SSA, the 10-item version of the CES-D has been found to be a valid, reliable tool to measure depression among the general Zulu, Xhosa, and coloured Afrikaans speaking populations in South Africa [31]. To our knowledge, however, the CES-D 10 specifically has not been validated across youth populations in any other SSA country.

The first validation of the full 20-item CES-D suggested a four-factor structure [1]. The four factors grouped together items into categories of 1) depressed affect, 2) positive affect, 3) somatic activity, and 4) interpersonal relations [17]. While this four-factor structure has been replicated in studies conducted among diverse populations in the US [21, 32, 33] and populations outside the US [20, 34], both three-factor [35–37] and two-factor solutions [23, 38, 39] have also been identified. In SSA, studies have found the original four-factor solution among HIV-infected adults in Uganda [24] and students in South Africa [8] but a two-factor solution among genocide survivors in Rwanda [25]. Validations of the CES-D 10, however, have typically found a two-factor structure representing positive and negative affect [26, 27, 29–31], while other studies have found a single-factor solution including one among adolescents in France [28] and among an Afrikaans speaking population in South Africa [31].

### CES-D correlates among youth

A variety of factors have been found to be associated with depressive symptomatology among youth, including individual and household-level characteristics as well as exposure to negative life events and the social environment. Female gender is one of the most consistent characteristics associated with depressive symptoms [20, 40, 41]. Other individual factors associated with depressive symptoms include increasing age, belonging to an ethnic minority group [41], and having lower self-esteem [40]. At the household level, lower socio-economic status (e.g. low adult education levels or low income) [41], poor physical or mental health of a parent [42], and family conflict or poor parent-child relationships [43–45] are linked to increased levels of youth depressive symptoms. Social environmental factors including the school environment (e.g. competition among pupils, control by teachers, and pressure to achieve) [40] and discrimination related to ethnicity [43] can also serve as a risk factor for depressive symptoms among young people. Lastly, young people are at greater risk of depression when exposed to negative life events such as problems in intimate relationships [46] including exposure to intimate partner violence [47].

The factors associated with depressive symptoms among youth in SSA generally follow the same pattern as elsewhere. For example, studies in SSA have frequently shown a gender disparity whereby females show more symptoms of depression [13, 14, 16]. Other individual risk factors include poor general health [13, 15] and behaviors such as substance misuse [16], heavy episodic drinking [9], and HIV risk behavior [10, 11]. At the household level, poverty and related conditions such as food insecurity are an important risk factor for depression [13]. Additionally, poor family environments and lack of social support are also related to higher levels of depressive symptoms for youth in SSA [9]. Lastly, traumatic experiences including forced sex, sexual partner violence, and having been abused as a child put youth at greater risk of depression [9, 10].

## Methods

### Data collection

This analysis uses data collected for impact evaluations of government cash transfer programs in rural areas of five countries: Kenya, Malawi, Tanzania, Zambia, and Zimbabwe. All analyses are conducted using pre-treatment (baseline) data, except for Kenya as explained below. These cash transfer programs were targeted at the household-level with the household head or caregiver receiving the transfer. A summary table of program characteristics for each country is provided in Additional file 1: Table S2.

All evaluations with the exception of Zimbabwe were designed as cluster-randomized controlled trials (cRCT) at the community or village level, with a random sample of program-eligible households interviewed in each community. Zimbabwe was designed as a district-matched case control evaluation. Household surveys were administered to either the transfer recipient or household head and covered a range of topics including consumption, food security, productive activities, and schooling and health of household members.

In addition, separate youth modules were administered to up to two or three youth per household according to a specified age range (varied by country), except for Tanzania where all youth in the specified age range were targeted to be interviewed. Topics included mental health, schooling, aspirations and expectations, sexual behaviors, risk preferences, and substance use. Youth were interviewed face-to-face in private settings by same-sex enumerators using the local language, and if privacy could not be assured, then enumerators were instructed to forgo the interviews. Informed consent was obtained from youth age 18 or above, and informed assent plus parental informed consent was obtained for youth aged younger than 18 years. Written consent was obtained in Kenya and Tanzania and verbal consent in Malawi, Zambia, and Zimbabwe where enumerators signed forms documenting that consent was asked and received. Verbal consent was given ethical approval in these settings as available data indicated that a large proportion of our main respondent sample would be illiterate and thus unable to read and sign a consent form. All studies were submitted to a national institutional review board (IRB) for ethical clearance, and in all cases except Tanzania, were submitted in parallel to international IRB for additional ethical review (see Additional file 1: Table S2).

### Individual country samples

The Kenyan youth sample come from the evaluation of Kenya’s Cash Transfer for Orphans and Vulnerable Children (CT-OVC). The youth module with the CES-D questionnaire was an addition to the 2011 endline survey. In order to ensure no program effects are captured in this analysis, we only use data from control households (those not receiving the cash transfer). Up to three Kenyan youth living in the household and aged 15–25 were administered the youth module (*N* = 651). The Malawian youth sample comes from the Malawi Social Cash Transfer Program (SCTP) impact evaluation. Up to three youth aged 13–19 were interviewed in each household at baseline in 2014 and included in this analysis (*N* = 2098). The Tanzanian youth sample comes from the impact evaluation of Tanzania’s Productive Social Safety Net (PSSN). Potentially all youth aged 14–28 from the households were interviewed at baseline in 2015 (*N* = 1357) and are included in this analysis. In Zambia, the youth sample comes from the impact evaluation of the Multiple Category Targeted Grant (MCTG). Up to two youth aged 13–17 per household were administered the youth module at baseline in 2011 and included in this analysis (*N* = 1982). The Zimbabwean youth sample is taken from baseline survey data of the evaluation of Harmonized Social Cash Transfer (HSCT) conducted in 2013. Up to three youth per household completed the youth module (*N* = 916).

All questionnaires and full country reports with additional sampling and evaluation details are available on the Transfer Project website (https://transfer.cpc.unc.edu). In each country, the CES-D was translated into local languages and field teams subsequently revised each translation for accuracy, interpretation and specificity in group settings during the training period. Local language translations for the CES-D 10 scales utilized here are provide in Additional file 1: Table S3.

### Measures

Our primary outcome measure is the CES-D 10 (hereafter referred to as CES-D in methodology and results) [26]. Each item of the CES-D is answered in reference to the past 7 days and on a one to four Likert scale [1 = rarely (< 1 day), 2 = some or a little of the time (1–2 days); 3 = occasionally or a moderate amount of time (3–4 days); 4 = most or all of the time (5–7 days)]. All ten items were summed and then rebased to zero for a CES-D scale score ranging from 0 to 30, where higher scores reflect greater depressive symptomology. A binary indicator was then created using a cutoff of 10 or more to be indicative of a youth exhibiting depressive symptoms. This cutoff is the most commonly used threshold and has been previously used in SSA [9, 11, 12, 15].

### Statistical analysis

#### Descriptive statistics and internal consistency

Descriptive summary statistics are provided for each country separately for the full youth sample and for the subsample of youth aged 18 years and under. We use these two samples because of additional data indicator availability (orphanhood) in the younger age group and to observe any heterogeneity in results, given the expectation that rates of depressive symptoms differ by age. We then examined average CES-D scores, individual item scores, and levels of depression for both groups. Internal consistency was assessed using Cronbach’s alphas.

#### Factor analysis

Exploratory and confirmatory factor analyses (EFA and CFA, respectively) were used to examine the factor structure and model fit of the CES-D in each of the study samples. An EFA with an orthogonal varimax rotation was first conducted to assess whether the factor structure and underlying relationships between the CES-D items were the same across samples [26]. We examined the performance of the CES-D scale with factor analysis, using five criteria (summarized in Additional file 1: Table S4) following previous studies validating the CES-D [38, 48, 49]: 1) Each factor must have an eigenvalue of equal to or greater than one, 2) Each item should load equal to or greater than 0.40 on the primary factor, 3) The difference between the item loading (see point 2) on the primary factor and other factors should be at least 0.2, 4) Each factor must have at least three items loading at 0.3 or higher, and 5) Factors must have a coefficient alpha greater than 0.7.

Second, we conduced CFA to confirm whether the two factor structure with latent factors for negative and positive affect showed good model fit for each country sample. We performed CFA using maximum likelihood methods with a diagonal covariance structure. Model fit was assessed using recommended indices including *χ*^2^, the root mean square error of approximation (RMSEA), comparative fit index (CFI), the Tucker-Lewis index (TLI), and the standardized root mean squared residual (SRMR) [50]. Thresholds that indicate good to excellent fit are values where RMSEA ≤0.07, CFI ≥ 0.90, TLI ≥ 0.95 and SRMR ≤0.08.

#### Measurement invariance

We then assessed configural, metric, and scalar measurement invariance across gender and age using multi-group CFA by sequentially estimating more constrained models. The first model tested configural variance by allowing all parameters to vary across groups. The second model constrained the factor loadings to be equal across groups to test metric invariance, and then the third model constrains both factor loadings and intercepts to test scalar invariance. We examined model fit using the CFA fit indices and invariance by comparing successively constrained models to the previous model using differences in chi-square and CFI.

#### Construct validity

Finally, to assess construct validity, we examined associations between household- and individual-level characteristics and the CES-D. We used multivariate linear regression models to examine associations both for the full sample and for youth aged 18 years or younger. Characteristics examined were motivated by the existing literature. For example, we hypothesized that increasing age, orphan status, and female gender are associated with increasing CES-D scores, while school enrolment and wealth would be protective.

We ran individual analyses by country and age group due to data availability differences by these components. Individual-level variables include gender, age (years), current enrollment or educational attainment (typically measured as completing secondary level), chronic illness (reported morbidity for three or more months in the past years), and orphan status (having lost both parents, measured only among the sample aged 18 and under). However, for the Tanzanian sample, chronic illness and orphan status were not collected. Household-level variables include monthly per-capita expenditure in local currency units (logged in multivariate analysis), for all samples except Tanzania for which we use a wealth index created through principal component analysis using household assets and dwelling characteristics to capture household economic status. For orphan status, a small number of observations were missing. We replaced missing indicators with the mode of the sample average and added a binary indicator to represent missingness to regressions as a covariate. This strategy resulted in 1–2% of the sample with replaced indicators for orphan status by country (see Table 1 for details). Geographic fixed effects for region or district of randomization stratification are included as appropriate by country, but not reported in Tables. Standard errors were adjusted for clustering at the community-level (the level of program randomization). Data were analyzed using Stata version 14.

**Table 1:** Summary statistics of background characteristics, by country

	(1)	(2)	(3)	(4)	(5)
	Zimbabwe	Zambia	Tanzania	Malawi	Kenya
	Mean	Mean	Mean	Mean	Mean
*Panel A: All youth*					
Male (%)	0.51	0.53	0.49	0.51	0.59
Age (years)	15.29	14.89	19.29	15.34	18.80
Chronically ill (3+ months in past year) (%)	0.04	0.01	–	0.07	0.06
Enrolled in school or completed secondary (%)	0.68	0.76	0.28	0.67	0.63
Monthly per capita expenditure (local currency)	24.92	42.34	0.08^1^	36,855	2503
*N*	918	1982	1189	2098	651
*Age range (years)*	13–19	13–17	14–28	13–19	15–25
*Panel B: Youth aged 18 and younger*					
Male (%)	0.50	0.53	0.54	0.51	0.57
Age (years)	15.06	14.89	15.75	15.12	16.54
Chronically ill (3+ months in past year) (%)	0.04	0.01	–	0.07	0.04
Orphan (both parents) (%)	0.43	0.64	–	0.45	0.75
Missing orphan status (%)	0.01	0.02	–	0.02	0.02
Enrolled in school (%)	0.68	0.77	0.45	0.68	0.77
Monthly per capita expenditure (local currency)	24.68	42.34	0.13^1^	36,789	2402
*N*	869	1982	611	1979	341
*Age range (years)*	13–18	13–17	14–18	13–18	15–18

Samples are taken from baseline surveys of cash transfer evaluations and include youth from poor and vulnerable rural households, with the exception of Kenya for which measures were collected at endline and include only the control group. ^1^ In Tanzania, a wealth index was used in place of monthly per capita expenditure, constructed via principle component analysis including household assets, dwelling characteristics. In Tanzania, chronic illness and orphan status were not collected

## Results

Table 1 describes the background characteristics of the five samples for the full sample (Panel A) and separately for youth aged 18 and younger (Panel B). For Zambia, the full sample is under 18, however Panels A and B differ because of some age-specific indicators included in the Panel B (e.g. orphan status). Across samples in Panel A, the proportion of males to females is comparable, expect for Kenya where males make up 59% of the full sample. The average age of participants is approximately 15 years in Malawi, Zambia, and Zimbabwe and approximately 19 years in Kenya and Tanzania where the age range of participants extends into the upper 20s. The large majority of youth are either enrolled in school or have completed secondary school across samples, except for in Tanzania, where only 28% of the full sample is enrolled in or has completed schooling. The younger subsamples in Panel B appear similar to the full samples across characteristics, although, schooling (enrollment only) increases to 45% for the Tanzanian sample. Orphanhood, an additional indicator for those 18 years and under, is high (over 40%) across each country with available data. A full three-fourths of Kenyan youth aged 18 and under are orphans, a result of Kenya targeting households with orphans and vulnerable children for their program.

Across both age group samples, the mean CES-D score ranged from 7.9 in Zambia to 11.8 in Tanzania (Table 2). Scores correspond to a low of 33% of youth in Zambia exhibiting depressive symptoms to a high of 64% in Tanzania. In all countries and samples, Cronbach’s alpha was greater than 0.70 for the CES-D scale with the exception of Tanzanian youth 18 years or younger (alpha = 0.67), indicating overall satisfactory internal reliability.

**Table 2:** Summary statistics of CES-D 10 outcome indicators including individual items, by country

	(1)	(2)	(3)	(4)	(5)
	Zimbabwe	Zambia	Tanzania	Malawi	Kenya
	mean	mean	mean	mean	mean
*Panel A: All youth (individual items (1)-(10) range: 1–4)*					
CES-D scale	8.73	7.89	11.76	9.95	8.61
Depressed (CESD≥10)	0.38	0.33	0.64	0.48	0.37
(1) Did you sleep well? (reverse coded)	1.42	1.80	2.18	1.72	1.47
(2) Were you happy? (reverse coded)	1.55	1.92	2.34	1.96	1.67
(3) Did you have trouble concentrating?	2.07	1.85	2.13	1.94	2.08
(4) Did you feel hopeful about the future? (reverse coded)	2.05	1.96	2.56	2.35	1.58
(5) Did you feel that everything you did was an effort?	2.67	2.33	2.53	2.33	2.81
(6) Did you feel lonely?	1.92	1.62	2.03	1.78	1.90
(7) Did you feel depressed?	1.89	1.51	2.29	1.93	1.74
(8) Did you feel that you could not get going?	1.77	1.63	2.08	2.41	1.83
(9) Were you bothered by things that don’t usually bother you?	1.68	1.61	1.73	1.77	1.82
(10) Did you feel fearful?	1.72	1.65	1.90	1.76	1.71
*N*	918	1982	1189	2098	651
*Alpha*	0.74	0.70	0.70	0.71	0.76
*Age range (years)*	13–19	13–17	14–28	13–19	15–25
*Panel B: Youth aged 18 and younger (individual items (1)-(10) range: 1–4)*					
CES-D scale	8.73	7.89	10.27	9.94	8.12
Depressed (CESD≥10)	0.38	0.33	0.53	0.47	0.35
(1) Did you sleep well? (reverse coded)	1.43	1.80	2.05	1.72	1.39
(2) Were you happy? (reverse coded)	1.55	1.92	2.15	1.96	1.63
(3) Did you have trouble concentrating?	2.08	1.85	1.90	1.94	2.02
(4) Did you feel hopeful about the future? (reverse coded)	2.05	1.96	2.52	2.35	1.48
(5) Did you feel that everything you did was an effort?	2.66	2.33	2.44	2.32	2.75
(6) Did you feel lonely?	1.90	1.62	1.90	1.78	1.84
(7) Did you feel depressed?	1.89	1.51	1.96	1.94	1.72
(8) Did you feel that you could not get going?	1.77	1.63	1.97	2.40	1.83
(9) Were you bothered by things that don’t usually bother you?	1.68	1.61	1.58	1.77	1.77
(10) Did you feel fearful?	1.73	1.65	1.80	1.77	1.69
*N*	869	1982	611	1979	341
*Alpha*	0.75	0.70	0.67	0.72	0.78
*Age range (years)*	13–18	13–17	14–18	13–18	15–18

CES-D scale ranges from 0 to 30; while Depressed is a binary outcome indicating a scale value higher or equal to 10. Samples are taken from baseline surveys of cash transfer evaluations and include youth from poor and vulnerable rural households, with the exception of Kenya for which measures were collected at endline and include only the control group

As part of the EFA, we found either two or three factors emerged across countries. Using Criteria 1 (eigenvalues of one or greater), Malawi and Zimbabwe had two factors while Zambia, Tanzania and Kenya each had three factors. However, a visual test of the slopes (“Scree test”), indicates that third factors are only marginally above or at one, and slopes flatten out between two to three factors (Additional file 1: Figure S1). Rotated factor loadings are displayed in Table 3. Countries consistently displayed strong validity for criteria (2), (3) and (4), greater than or equal to 0.40 loading on the primary factor, no cross-loading (differences are at least 0.20 between factors for each item) and no trivial factors (at least three items loading ≥0.30 for each factor). The exceptions are cross-loadings for ‘hopeful’ and ‘concentrate’ in Kenya and ‘lonely’ and ‘depressed’ in Tanzania. Loadings of ‘hopeful’ were also generally lower on the primary factor (including < 0.40 on in Tanzania), a result also found in South Africa [31]. The item ‘effort’ was the only item that loaded on the third factors, indicating these third factors performed particularly poor (alphas range from 0.00–0.18). The individual alphas for other two factors, however, were also lower than expected (below 0.49). Nevertheless, in the original validation of the CES-D, Radloff (1977) argues against overemphasis of the individual factors due to high internal consistency of the overall scale, which we also find in our samples [17].

**Table 3:** Rotated factor analysis of CES-D 10 items (all youth sample)

	Zimbabwe		Zambia			Tanzania			Malawi		Kenya		
	(1)	(2)	(3)	(4)	(5)	(6)	(7)	(8)	(9)	(10)	(11)	(12)	(13)
*All items range from 1 to 4*	Factor 1	Factor 2	Factor 1	Factor 2	Factor 3	Factor 1	Factor 2	Factor 3	Factor 1	Factor 2	Factor 1	Factor 2	Factor 3
(1) Did you sleep well? (reverse coded)	0.12	**0.71**	0.12	**0.85**	−0.05	0.07	**0.76**	−0.11	0.10	**0.74**	0.16	**0.81**	0.00
(2) Were you happy? (reverse coded)	0.27	**0.72**	0.09	**0.86**	0.05	0.03	**0.77**	−0.29	0.14	**0.78**	0.06	**0.82**	0.08
(3) Did you have trouble concentrating?	**0.56**	−0.11	0.32	0.17	**0.56**	0.37	**0.51**	0.25	**0.63**	0.08	**0.40**	**0.41**	0.29
(4) Did you feel hopeful about the future? (reverse coded)	0.09	**0.65**	0.11	**0.41**	−0.62	0.06	0.24	− 0.74	− 0.16	**0.57**	0.27	**0.42**	−0.49
(5) Did you feel that everything you did was an effort?	**0.37**	−0.46	0.11	0.06	**0.81**	0.01	−0.02	**0.80**	**0.64**	0.02	0.13	0.12	**0.77**
(6) Did you feel lonely?	**0.72**	0.14	**0.64**	0.03	0.14	**0.57**	0.39	0.12	**0.63**	0.07	**0.65**	0.14	0.32
(7) Did you feel depressed?	**0.76**	0.14	**0.77**	0.10	0.04	**0.58**	0.52	0.14	**0.59**	0.25	**0.67**	0.18	0.28
(8) Did you feel that you could not get going?	**0.74**	0.12	**0.75**	0.12	0.03	**0.79**	−0.04	−0.05	**0.60**	0.10	**0.72**	0.11	−0.06
(9) Were you bothered by things that don’t usually bother you?	**0.70**	0.16	**0.73**	0.14	0.07	**0.75**	0.06	−0.10	**0.70**	0.03	**0.74**	0.06	−0.05
(10) Did you feel fearful?	**0.66**	0.09	**0.59**	0.22	0.18	**0.57**	0.29	0.00	**0.68**	0.07	0.67	0.23	−0.04
*N*	918	918	1982	1982	1982	1189	1189	1189	2098	2098	651	651	651
*Alpha*	0.49	0.21	0.46	0.38	0.18	0.39	0.38	0.00	0.46	0.30	0.43	0.32	0.15

Samples are taken from baseline surveys of cash transfer evaluations and include youth from poor and vulnerable rural households, with the exception of Kenya for which measures were collected at endline and include only the control group

The highest factor loadings for each item are in bold

Given the results of the EFA, the two-factor solution appears to fit the data better. Therefore, we ran CFA on the two factor model where Factor 1 consisted of ‘sleep well’, ‘happy’, and ‘hopeful’ while Factor 2 consisted of ‘concentrate’, ‘effort’, ‘lonely’, ‘depressed’, ‘get going’, ‘bothered’, and ‘fearful’. Overall, this two-factor solution showed great fit for Malawi, Zimbabwe, and Kenya (Table 4). For each of these countries, the RMSEA ≤ 0.06, CFI ≥ 0.93, TLI ≥ 0.91, and SRMR ≤ 0.05. The two factor solution was not as strong, but still showed good fit for Zambia (RMSEA = 0.08, TLI = 0.85, CFI = 0.89, SRMR = 0.06) and Tanzania (RMSEA = 0.08, TLI = 0.84, CFI = 0.88, SRMR = 0.06). For the pooled sample, we also find highly acceptable model fit (RMSEA = 0.06, TLI = 0.91, CFI = 0.93, SRMR = 0.04). All *χ*^2^ values were significant at *p* < 0.001, however these tests are very sensitive to large sample sizes, and as such, not useful for comparing fit in this analysis.

**Table 4:** Conformatory Factor Analysis (CFA) fit indices (all youth sample)

Sample	*χ* ^2^	df	*p*-value	RMSEA	RMSEA 90% CI lower	RMSEA 90% CI upper	TLI	CFI	SRMR
Pooled	902.36	34	0.00	0.06	0.06	0.06	0.91	0.93	0.04
Zimbabwe	147.10	34	0.00	0.06	0.05	0.07	0.93	0.94	0.05
Zambia	496.17	34	0.00	0.08	0.08	0.09	0.85	0.89	0.06
Tanzania	312.53	34	0.00	0.08	0.07	0.09	0.84	0.88	0.06
Malawi	213.40	34	0.00	0.05	0.04	0.06	0.93	0.95	0.04
Kenya	120.03	34	0.00	0.06	0.05	0.07	0.91	0.93	0.04

*χ*^2^ chi-square, *df* degree of freedom, *RMSEA* root mean square error of approximation, *CI* confidence interval, *TLI* Tucker-Lewis index, *CFI* comparative fit index, *SRMR* standardized root mean squared residual. Pooled sample includes all country samples

Next, we examined measurement invariances using the pooled sample across gender, age groups (> 18 years and ≤ 18), and by country (Table 5). For model identification purposes, means were set to 0 in both groups and variances were set to 1. Across multi-group CFA models for gender and age, fit indices indicate good to acceptable model fit and invariance appears to be upheld. For gender, we find that full invariance is supported (change in CFIs was < 0.01) suggesting that males and females answer scale items in the same way. For age, configural and metric invariance was supported (0.01 difference in CFIs) while scalar invariance is not supported indicating averages for older youth (> 18 years) may be systematically different than younger youth (≤ 18 years). Across country samples, fit indices are good for the first model indicating configural variance but neither metric nor scalar measurement invariance is supported. Therefore, there are likely systematic differences in the way CES-D was answered across countries, possibly due to cultural differences, upper bounds of age ranges per country or the ability of local language translation to capture consistent and specific item concepts.

**Table 5:** Measurement invariance by country, gender and age group

	*χ* ^2^	df	p-value	RMSEA	RMSEA 90% lower	RMSEA 90% upper	TLI	CFI	SRMR	Reference Model	∆x^2^	∆CFI
Gender												
1.Configural	941.63	68	0.00	0.06	0.06	0.06	0.91	0.93	0.04	–	–	–
2.Metric	966.62	78	0.00	0.06	0.05	0.06	0.92	0.93	0.05	1	24.99	0.00
3.Scalar	1007.03	88	0.00	0.06	0.05	0.06	0.93	0.93	0.05	2	40.41	0.00
Age group (> 18 years vs ≤ 18 years)												
1.Configural	962.39	68	0.00	0.06	0.06	0.07	0.91	0.93	0.05	–	–	–
2.Metric	1059.65	78	0.00	0.06	0.06	0.06	0.91	0.92	0.06	1	97.26	−0.01
3.Scalar	1326.31	88	0.00	0.06	0.06	0.07	0.90	0.90	0.07	2	266.66	−0.02
Country												
1.Configural	1289.23	170	0.00	0.07	0.07	0.07	0.89	0.91	0.05	–	–	–
2.Metric	1840.18	210	0.00	0.08	0.07	0.08	0.87	0.87	0.07	1	550.95	−0.04
3.Scalar	4102.85	250	0.00	0.11	0.10	0.11	0.73	0.70	0.07	2	2262.67	−0.17

*χ*^2^ chi-square, *df* degree of freedom, *RMSEA* root mean square error of approximation, *CI* confidence interval, *TLI* Tucker-Lewis index, *CFI* comparative fit index, *SRMR* standardized root mean squared residual. Measurment invariance by groups estimated with pooled sample

Table 6 displays the results from OLS regression models for the relationship between the CES-D scale and individual and household determinants. Relationships are similar for the full sample (Panel A) and the 18 years and younger sample (Panel B). Of the individual determinants, CES-D scores increase with age, significant in all samples but Malawi. Either being enrolled in school and/or having completed secondary has a protective relationship for youth, although not all relationships across countries and samples are statistically significant. The relationship between CES-D and gender is less conclusive in our samples, with males exhibiting significantly lower CES-D scores in Tanzania but higher scores in Kenya as compared to females. This can also be seen visually in Additional file 1: Figure S2 with country level kernel density graphs of CES-D scores by gender for the 18 and younger samples. In only one case (Zambia), is orphanhood negatively associated with CES-D scores as originally hypothesized. Chronic illness was not associated with CES-D scores, however as shown in Table 1, the percentage of youth reporting chronic illness is very low across all samples (from 1 % in Zambia to 7% in Malawi). Increasing per capita household expenditures were protective, particularly in Zimbabwe and Zambia were measures are highly statistically significant.

**Table 6:** Ordinary Least Squares multivariate regression examining assocations with CES-D scale (range: 0–30)

	(1)	(2)	(3)	(4)	(5)
	Zimbabwe	Zambia	Tanzania	Malawi	Kenya
*Panel A: All youth*					
Male	− 0.35 (0.37)	0.14 (0.23)	−1.15*** (0.38)	− 0.12 (0.44)	1.09** (0.41)
Age (years)	0.18** (0.08)	0.22*** (0.07)	0.33*** (0.04)	0.07 (0.08)	0.19* (0.10)
Chronically ill (3+ months in past year)	1.13 (0.96)	0.48 (1.40)	––	0.15 (0.50)	0.71 (1.02)
Enrolled in school or completed secondary	−0.85** (0.37)	−0.58** (0.29)	−0.37 (0.44)	−1.02** (0.41)	−0.20 (0.60)
Log per capita monthly expenditure	−1.28*** (0.41)	−0.82*** (0.20)	−0.18 (0.18)	− 0.59** (0.24)	0.50 (0.59)
Constant	12.19*** (1.85)	8.57*** (1.47)	5.89*** (1.11)	15.67*** (2.54)	1.01 (5.40)
*N*	916	1982	1189	2098	651
*R-squared*	0.06	0.04	0.09	0.01	0.03
*Age range (years)*	13–19	13–17	14–28	13–19	15–25
*Panel B:18 years and younger*					
Male	−0.28 (0.37)	0.13 (0.23)	−1.08** (0.48)	−0.06 (0.45)	1.51*** (0.51)
Age	0.24** (0.11)	0.22*** (0.07)	0.44** (0.17)	0.10 (0.11)	0.52 (0.38)
Chronically ill (3+ months in past year)	1.09 (0.99)	0.56 (1.39)	– –	0.16 (0.54)	2.35 (1.95)
Orphan (both parents)	0.15 (0.35)	0.49* (0.28)	– –	0.30 (0.31)	0.72 (0.77)
Missing orphan status	0.15 (1.66)	1.05 (0.96)	– –	−0.50 (0.73)	2.95 (4.22)
Enrolled in school	−0.84** (0.39)	−0.65** (0.28)	− 0.41 (0.43)	−0.99** (0.44)	−1.21 (0.93)
Log per capita monthly expenditure	−1.37*** (0.42)	−0.82*** (0.20)	−0.24 (0.22)	− 0.56** (0.23)	1.03 (0.67)
Constant	11.42*** (2.20)	8.29*** (1.48)	3.56 (2.76)	14.78*** (2.82)	−8.40 (9.98)
*N*	866	1982	611	1979	341
*R-squared*	0.06	0.04	0.04	0.01	0.08
*Age range (years)*	13–18	13–17	14–18	13–18	15–18

Robust standard errors in parentheses; *** *p* < 0.01, ** *p* < 0.05, * *p* < 0.1. Geographic fixed effects for region or district of randomization stratification are included as appropriate by country but not reported

Samples are taken from baseline surveys of cash transfer evaluations and include youth from poor and vulnerable rural households, with the exception of Kenya for which measures were collected at endline and include only the control group. In Tanzania, a wealth index was used in place of monthly per capita expenditure, constructed via principle component analysis including household assets, dwelling characteristics. In Tanzania, chronic illness and orphan status were not collected

## Discussion

As poor mental health is a leading cause of death and disability-adjusted life years (DALYs) among young people globally, more evidence is needed to understand effective initiatives to improve youth mental health and well-being. Before such evidence can be generated, standard tools for measurement of mental health, including depressive symptoms, need to be validated among diverse youth populations. Using data from five countries, this study is the first to examine the psychometric properties of the CES-D 10 among young people in rural, poor households in SSA. We find positive evidence to support the use of this measure in such populations.

Our analyses reveal that the CES-D 10 performs well across samples and that relationships between the scale and characteristics largely aligned with hypotheses driven by the literature on the determinants of youth depression. EFA results and factor loadings pointed to a two-factor solution for the CES-D 10 as the most likely factor structure. In Tanzania, Zambia, and Kenya, the item ‘everything was an effort’ loaded differently, suggesting the expression of depressive symptoms may include an additional somatic element in these settings. Similar to other studies on the CES-D 10, the main factors fit into positive and negative affect [26, 27, 29–31] with positive affect including ‘happy’, ‘hopeful’, and ‘sleep well’, the three reverse coded questions. Similar to another validation study in South Africa, ‘hopeful’ loaded lower compared to other positive affect items indicating that ‘hopeful’ may not perfectly align with the conceptualization of positive affect [31] *.*CFA results indicate good model fit for the two-factor model for most countries and the pooled sample, although fit for Tanzania and Zambia are not as strong. Multi-group CFA results in the pooled sample also indicated strong invariance of CES-D across gender, but weaker invariance across age and country samples.

Patterns of background characteristics associated with the CES-D were similar in the entire age range and adolescent samples, and were generally consistent with the existing literature. For example, gender and age tend to be two of the most salient characteristics associated with CES-D. In our samples, we also find that increasing age is associated with increased depressive symptoms, however the findings related to gender are mixed. In two of the countries (Tanzania and Malawi), we found associations in the expected direction, with males reporting lower scores. However, in Zimbabwe and Zambia we found no association with gender and in Kenya, males reported higher CES-D scores. The Kenyan sample is unique in its skewed gender distribution (almost 60% male), and this reflects the composition of households targeted for the cash transfer program from which the sample was drawn, namely, those supporting orphans and vulnerable children. It is not known why there were fewer female adolescents in these households, however this systematic difference may contribute to the unexpected direction of the relationship between gender and CES-D in this sample.

We also find that school enrolment and wealth are protective in three out of the five countries studied. We did not find a protective relationship with school enrolment in the Tanzanian sample, however, school enrolment was the lowest across all countries studied and average age was the highest, which may have contributed to the resulting lack of association. Orphan status was generally not associated with CES-D, with the exception of one country (Zambia), where the association was in the hypothesized direction. In general, orphan rates were high in these samples (ranging from 43 to 75% among adolescents), reflecting the targeting of the cash transfer programs to labor-constrained households (who are often elderly relatives caring for orphaned children, with a “missing” generation of able-bodied adults). This detail, combined with the fact that these samples are quite homogenous in terms of high poverty, food insecurity, and limited access to labor market opportunities, may indicate that the CES-D may not be sensitive enough to detect unique distress resulting from orphanhood status, above and beyond the chronic stressors that these adolescents face.

Between one-third and two-thirds of our samples display depressive symptoms as determined by recommended cut-offs, and although the CES-D 10 is not a diagnostic tool, such levels indicate a high burden of psychosocial issues in these youth populations. Comparing our results to other studies using the CES-D in SSA, we find that levels in Zambia, Zimbabwe, and Kenya (where the percentage exhibiting depressive symptoms according to the cutoff is less than 40%) are on par with the findings from a number of studies (see Additional file 1: Table S2) [9, 10, 12, 15]. However, in both Malawi and Tanzania, our results are more striking with over 50% of each sample displaying depressive symptoms. According to a recent review by Sweetland, Belkin, and Verdeli (2014), the use of brief psychiatric instruments in SSA is not without significant hurdles [5]. Cultural differences in the expression or manifestation of depressive symptoms can make it hard to simply translate instruments and capture the conceptual equivalent of the disorder (measurement variance across countries we studied is a likely indication of this). Previous work in Tanzania, for instance, has shown that the experience and expression of depression differs from western cultures, particularly due to the absence of depressed mood [5]. In this way, the high rate of depressive symptoms displayed in the Tanzanian sample may partly reflect a lack of conceptual equivalence and adjusting the CES-D scale items or cut-offs may be warranted.

Nevertheless, it is important to recognize that the youth populations used in this study are among the poorest and most vulnerable in the region. The intensity and persistence of adverse conditions (including high rates of orphanhood, exposure to violence and generalized HIV prevalence affecting caregivers and youth themselves) during childhood likely puts them at even greater risk of psychosocial problems than their peers in the same settings. Moreover, adolescence and young adulthood is a particularly vulnerable time for the development of depressive disorders and even in high-income countries, the prevalence of disorders among youth hovers around 20% [4]. In general, there is a lack of evidence on the burden of mental health problems among young people in SSA, thus our findings add to this limited evidence base.

Strengths of this study include large sample sizes, diverse geographic locations, and inclusion of data on household-level socioeconomic indicators. However, there are some limitations to this study. First, the samples come from impact evaluations of poverty-targeted cash transfer programms, which means youth came from extreme poor and rural households. This may limit generalizability of findings in the region. Nonetheless, by demonstrating good performance of the CES-D in a population where we may expect challenges to implementing standard scales, bolsters expected validity among other youth populations in these countries.

Another limitation is that implementation of the tool necessitated translation into local languages, which often lack diversity in vocabulary to adequately allow nuance and differentiate concepts of individual scale items. Additionally, because these data were collected in the context of larger impact evaluations, the questionnaires were not designed with validation of the CES-D scale as an objective. Therefore, related measures of mental health were not collected, which would have been helpful for testing construct validity of the CES-D. Finally, the fact that some reverse-scored items hung together in the factor analysis (in Zimbabwe, Zambia, Kenya) suggests that our data may suffer from some degree of reporting bias. Other studies have also reported this same phenomenon [31] and so these reverse coded items may tend to be somewhat confusing to participants. Nevertheless, other evidence described above suggests that the CES-D generally performed well in these populations, supporting its expanded use.

## Conclusions

In summary, this study provides novel evidence supporting the use of the CESD 10 among youth in SSA. This tool can be used in future efforts to study dynamics of depressive symptoms in this population, as well as effectiveness of policies and interventions to improve the mental health of adolescents in SSA. Our results are suggestive that the burden of mental illness is very high among the most poor and vulnerable youth populations in SSA. However, structural interventions, including policies and initiatives which promote school enrollment and economic strengthening may have the potential to improve adolescent mental health. We recommend further investigations in this area to understand the protective and promotive effects of such interventions on youth mental health and psychosocial development.

## Additional file

Additional file 1: The additional figures and tables contained in this document explains the background literature we cite and our data and results in more detail. The file contains the following tables and figures, which are cited in the text with the corresponding figure or table number. **Figure S1.** Plotted test of eigenvalues across countries from full youth samples. **Figure S2.** Kernel density graphs of CES-D 10 scores for 18 years and under samples by individual country. **Table S1.** Studies in sub-Saharan Africa using the CES-D among or including youth populations (alphabetical by author). **Table S2**. Summary of cash transfer program and evaluation characteristics. **Table S3**. Questionnaire translations for CES-D 10 in local languages. **Table S4**. Summary of criteria for reliability and validity assessment of CES-D scale among the full sample. (DOCX 201 kb)

## Abbreviations

CES-D 10: Center for epidemiological studies depression 10-item scale
CES-D: Center for epidemiological studies depression scale
CFA: Confirmatory factor analysis
CFI: Comparative fit index
CI: Confidence interval
cRCT: Cluster-randomized controlled trial
CT-OVC: Cash transfer for orphans and vulnerable children
DALYs: Death and disability-adjusted life years
df: Degrees of freedom
EFA: Exploratory factor analysis
HIV: Human immunodeficicy virus
HSCT: Harmonized social cash transfer
IRB: Institutional review board
LMIC: Low- and middle-income countries
MCTG: Multiple category targeted grant
OLS: Ordinary least squares
PSSN: Productive social safety net
RMSEA: Root mean square error of approximation
SCTP: Social cash transfer program
SRMR: Standardized root mean squared residual
SSA: Sub-Saharan Africa
TLI: Tucker-lewis index
US: United States
