# Supplementary material for: Examination of performance of the Center for Epidemiologic Studies Depression Scale Short Form 10 among African youth in poor, rural households
Source: BMC Psychiatry. 2018 Jun 18;18:201. doi: 10.1186/s12888-018-1774-z (PMC6006708; doi:10.1186/s12888-018-1774-z)
Supplement: Supplementary file 1 — The additional figures and tables contained in this document explains the background literature we cite and our data and results in more detail. The file contains the following tables and figures, which are cited in the text with the corresponding figure or table number. Figure S1. Plotted test of eigenvalues across countries from full youth samples. Figure S2. Kernel density graphs of CES-D 10 scores for 18 years and under samples by individual country. Table S1. Studies in sub-Saharan Africa using the CES-D among or including youth populations (alphabetical by author). Table S2. Summary of cash transfer program and evaluation characteristics. Table S3. Questionnaire translations for CES-D 10 in local languages. Table S4. Summary of criteria for reliability and validity assessment of CES-D scale among the full sample. (DOCX 201 kb) [file 12888_2018_1774_MOESM1_ESM.docx]

**Online appendix material**

**Figure A1. Plotted test of eigenvalues across countries from full youth samples**

Notes: Plots based on “Scree test” suggested in Atkins (2014)

**Figure A2. Kernel density graphs of CES-D 10 scores for 18 years and under samples by individual country**

**Table A1. Studies in sub-Saharan Africa using the CES-D among or including youth populations (alphabetical by author)**

| **Reference** | **Aim of study** | **Sample** | **Location** | **Depression** | **Correlates of depressive symptoms** | **Internal**  **Reliability**  **(Cronbach’s Alpha)** |
| --- | --- | --- | --- | --- | --- | --- |
| Asante & Andoh-Arthur [2015] | Cross-sectional study to assess prevalence and determinants of depressive symptoms. | 270 university students [average age 22 years] from the Department of Psychology were conveniently sampled. | Accra, Ghana | 39.2% scored above a cut-off point at 10 using the 10-item CES-D. | Lack of support, heaving episodic drinking, forced sex, physical and sexual assault as a child, and having been beaten by a sexual partner were associated with depressive symptoms. | 0.72 |
| Baron et al. [2017] | Cross-sectional study to establish the reliability and validity of the CES-D-10 among the general adult Zulu, Xhosa and Afrikaans speaking populations in South Africa. | Stratified random samples of Xhosa, Afrikaans and Zulu-speaking participants aged 15 years or older (N = 944)  (Very few adolescents were recruited to the study and almost none reported depression so results are for the full sample). | Cape Town Metro and Ethekwini districts, South Africa | 6.6% of Zulu, 18.0% of Afrikaans and 6.9% of Zulu samples were diagnosed with depression  using MINI diagnosis. The most appropriate CES-D 10 cut-offs were found to be 12, 11 and 13, respectively. | Socio-demographic measures associated with depression included female gender, older age, being divorced or widowed and retired were associated with depression. | 0.69-0.89 |
| Brown et. al. [2009] | Quasi-experimental study to test a mentorship program for youth-headed households and the effect on psychosocial outcomes among the youth. | 692 youth heads of households in intervention and control villages were interviewed [12-24 years] at baseline. At follow-up, 593 individuals [14-27 years] were included. | South-western Rwanda | Average CES-D score, 23.3 [control group] using the 20-item CES-D. | Mentorship program participation was associated with lower levels of depressive symptoms. Being female, a higher education, not living alone, having a parent killed in the genocide, poor health, having fewer assets and eating only one meal per day were associated with more depressive symptoms. | 0.86 |
| Kilburn et. al [2015] | Randomized controlled trial to study the impact of a cash transfer program on mental health outcomes among youth. | 1960 young people [15-24 years] were interviewed 4 years after the start of the program. | Kenya | 32% in the intervention group and 37% in the control group scored above a cut-off point of 10 using the 10-item CES-D. | The cash transfer reduced the odds of showing depressive symptoms. In addition, higher age and living in Nairobi were associated with higher odds of depression and no illness/injury in the past 4 weeks were associated with lower odds. | Not reported in text |
| Mukabutera et. al [2013] | Cross-sectional study with the aim to investigate psychosocial outcomes [including depression] and socio-economic status and quality of mentoring relationships. | 201 youth heads of households [11-24 years] and participating in an adult mentoring program were sampled. | Bugasera, Rwanda | The 20-item CES-D was used. | Being female, location of the household and water from river, rain or stream as main water source were associated with depression. | 0.87 |
| Nduna et. al. [2013] | Cross-sectional study with the aim to explore determinants of depressive symptoms in South African youth. | 1415 women and 1368 men [15–26 years] enrolled in an evaluation study of an HIV intervention were sampled based on their willingness to participate. | Eastern Cape Province, South Africa | Prevalence of depressive symptoms was 20.5% in women and 13.5% in men using a cut-off at 16 using the 20-item CES-D. | Factors associated with higher levels of depressive symptoms: Being female, childhood adversity, sexual violence and substance misuse. Among women only: intimate partner violence and lower perceptions of community cohesion. Among men only: a mother’s death and relationship conflict. | 0.90 for women;  0.91 for men |
| Neese et al. [2013] | Cross-sectional study with the aim to explore links between stress, coping strategies, depression and somatic complaints. | 299 adolescents [11-19 years] were recruited from a boarding secondary school. | Zambia | 37% scored above a cut-off of 28 using the 20-item CES-D. | Higher levels of perceived stress, and more coping were associated with increased depressive symptoms. | Not reported in text |
| Othieno et. al. [2015] | Cross-sectional study with the aim to describe links between risky sexual behaviour, depressive symptoms and socio-demographic characteristics | 923 [525 males and 365 females] undergraduate students with a mean age of 23 years were sampled. | Nairobi, Kenya | 41.33% scored above a cut-off point of 10 using the 10-item CES-D. | Tobacco use, positive history of HIV or other Sexual Transmitted Infection (STI), experience of traumatic event, such as abuse, as a child were associated with depression. | Not reported in text |
| Otwombe et. al. [2015] | Cross-sectional study with the aim to describe socio-demographic characteristics and health seeking behaviours. | 830 adolescents [14-19 years] were included in the study. | Soweto, South Africa | 48.1% scored above a cut-off point of 24 using the 20-item CES-D. | Not reported | 0.81 |
| Peltzer et al. [2013] | Cross-sectional study with the aim to explore links between mental health, childhood abuse and HIV sexual risk behaviour | 824 university students [50% men and 50% women] with a mean age of 23.7 years. | Abidjan, Ivory Coast | 17.6% scored above a cut-off point of 10 using the 10-item CES-D. | HIV risk behaviour was found to be associated with depression. | 0.72 |
| Pengpid et al. [2013] | Cross-sectional study to explore assocaitons between mental health, substance use and HIV sexual risk behaviour | 722 undergraduate university students [mean age = 21.7] were recruited from University of Limpopo Medical University of Southern Africa | Limpopo, South Africa | 39.5% showed depressive symptoms above a cut-off point of 10 using the 10-item CES-D. | Among men: intimate partner violence, forced sex and physical abuse as a child. Among women: sexual partner violence. | 0.72 |
| Pretorius [1991] | Cross-sectional study to determine the validity and reliability of the CES-D scale among black South African students. | 450 undergraduate students in psychology [19-53 years, mean age=24] from the University of the Western Cape. | Cape Town, South Africa | Average CES-D score 15.0 among men and 17.1 among women using the 20-item CES-D. | Being female, coming from a rural area and being a part-time student. | 0.90 |
|  |  |  |  |  |  |  |

**Table A2. Summary of cash transfer program and evaluation characteristics**

| **Country** | **Program** | **Targeting** | **Data collection** | **Ethical (IRB) Approval** |
| --- | --- | --- | --- | --- |
| Kenya | Cash Transfer for Orphans and Vulnerable Children  (CT-OVC) | Ultra-poor households with at least one orphan or vulnerable child (OVC) under the age of 18 residing in the household | Baseline data were collected in 2007 across seven districts (Homa Bay, Garissa, Kisumu, Kwale, Migori, Nairobi and Suba), however the youth module with the CES-D scale was an addition to the 2011 endline survey. Out of the 2,759 households that were interviewed at baseline; 2,255 were interviewed again at endline. | The University of North Carolina at Chapel Hill IRB and the Kenya Medical Research Institute Ethics Review Committee |
| Malawi | Social Cash Transfer Program (SCTP) | Ultra-poor, labor-constrained households (those with a dependency ratio higher than three) | Baseline data were collected in 2014 and include a total of 3,531 households in Salima and Mangochi districts. | The University of North Carolina at Chapel Hill IRB, Malawi’s National Commission for Science and Technology (NCST), and National Committee for Research in Social Sciences and Humanities |
| Tanzania | Productive Social Safety Net (PSSN) | Poor households living below the food poverty line, as determined by community-based targeting, and verified by a proxy means test | Baseline data were collected in 2015 in 84 communities within eight districts of mainland Tanzania (Misungwi, Kahama, Kilola, Kisarawe, Handeni, Mbogwe, Itilima, Uyui). The sample includes a total of 801 households, all of which include resident youth aged 14 to 28 years. | Tanzania’s Commission for Science and Technology (COSTECH) |
| Zambia | Multiple Category Targeted Grant (MCTG) | Households with a disabled member, or other vulnerable households such as those with a female or elderly head keeping orphans | Baseline data were collected in 2011 in 92 communities from the Luwingu and Serenje districts. The baseline sample includes 3,078 households. | American Institute for Research IRB and the University of Zambia’s Research Ethics Committee |
| Zimbabwe | Harmonized Social Cash Transfer (HSCT) | Food-poor and labor-constrained households (no able bodied prime-age member or dependency ratio of three or above, or special cases) | Baseline data were collected in 2013 six districts (Binga, Chiredzi, Hwange, Mudzi, Mwenzi and Uzumba-Maramba-Pfungwe (UMP)), within 90 wards, for a total of 3,063 households. | American Institute for Research IRB and the Medical Research Council of Zimbabwe |

**Table A3. Questionnaire translations for CES-D 10 in local languages**

|  | **English** | **Bemba [Zambia]** | **Chichewa [Malawi]** | **Shona [Zimbabwe]** | **Swahili [Kenya/Tanzania]** |
| --- | --- | --- | --- | --- | --- |
|  | Reference period is previous 7 days [one week] |  | Pa masiku 7 apitawa ndi masiku angati amene | Wakarara zvakanaka here? | Kipindi husika ni siku saba zilizopita [ wiki moja] |
| (1) | How often did you sleep well? | Munshiku 7 ishapita Kunuma, ninshiku shinga isho mwalelepo bwino? | Unagona bwino? | Wakaraa zvankanaka here? | Kwa siku saba zilizopita ni mara ngapi ulilala vizuri |
| (2) | How often were you happy? | Munshiku 7 ishapita kunuma, ninshiku shinga isho mwalipo abansansa? | Unali osangalala? | Wanga uchifara here? | Mara ngapi ulikuwa na furaha? |
| (3) | How often did you have trouble concentrating? | Munshiku 7 ishapita kunuma, ninshiku shinga isho mwakwetepo ubwafya bwa kutontontonkaya pachintu chimo? | Unali ndimavuto kutsatila zinthu mwachidwi? | Waive nedambudziko here rekuti pfungwa dzive pamwechete? | Mara ngapi umekuwa unashughuliki na matatizo? |
| (4) | How often do you feel hopeful about the future? | Munshiku 7 ishapita kunuma, ninshiku shinga isho tamwali abakusakamana pafintu ifyakuntanshi mumweo wenu? | Unali ndi chiyembekezo chabwino cha tsogolo? | Wanga une tariro yakanaka here mune remangwana? | Mara ngapi umekuwa na matumaini kuhusu wakati ujao? |
| (5) | How often did you feel that everything you did was an effort? | Munshiku 7 ishapita kunuma, ninshiku shinga isho mwaumfwile ati fyonse ifyo mwachitile fyali fyakutulukusha? | Kuti chilichonse umapanga unavutikila? | Wanga uchinzwa here kuti zvese zvawanga uchiita ndezvekushingaira? | Mara ngapi umejisikia kwamba kila kitu ulichofanya ni bidii? |
| (6) | How often did you feel lonely? | Munshiku 7 ishapita Kunuma, ninshiku shinga isho mwaumfwile abankumba bulili? | Unali osungulumwa? | Wanga uchisurukirwa here? | Mara ngapi unajisikia mpweke? |
| (7) | How often did you feel depressed? | Munshiku 7 ishapita Kunuma, ninshiku shinga isho mwaumfwile abatitikishiwa? | Unali okhumudwa? | Wanga uchinzwa kutambudzika mumoyo nemupfungwa here? | Mara ngapi unajisikia msongo wa mawazo? |
| (8) | How often did you feel that you ould not ‘get going’? | Munshiku 7 ishapita Kunuma, ninshiku shinga isho mwaumfwile ati teti mukwanishe ukutwalilila? | Unaona kuti zinthu sizikuyenda? | Wanga uchinzwa here kuti zvinhu hazvisi kufamba? | Mara ngapi unajisikia kuwa huwezi kujihamasisha mwenyewe kufanya kile kitu unachotaka kukifanya? |
| (9) | How often were you bothered by things that don’t usually bother you? | Munshiku 7 ishapita Kunuma, ninshiku shinga isho mwali abakusakamikwa nefintu ifishimisakamika? | Unasautsidwa ndi zinthu zimene sizimakusautsa nthawi zonse? | Wanga uchishungurudzika here nezvinhu zvisingawanzo kushungurudza? | Mara ngapi unasumbuliwa na vitu ambavyo kwa kawaida huwa havikusumbui? |
| (10) | How often did you feel fearful? | Munshiku 7 ishapita Kunuma, ninshiku shinga elyo mwali aba mwenso? | Unali ndi mantha? | Wanga uchinzwa kutya here? | Mara ngapi unajisikia woga? |

**Table A4 Summary of criteria for reliability and validity assessment of CES-D scale among the full sample**

| *Criterion* | | Zimbabwe | Zambia | Tanzania | Malawi | Kenya |
| --- | --- | --- | --- | --- | --- | --- |
| (1a) | Each factor with eigenvalue ≥1 should be rotated (number of qualifying factors) | 2 factors | 3 factors | 3 factors | 2 factors | 3 factors |
| (2) | Each item should load ≥0.40 on the primary factor | All items meet criteria, except effort | All items meet criteria | All items meet criteria, except effort and sleep | All items meet criteria | All items meet criteria except concentrate and effort |
| (3) | No cross-loading (a difference of at least 0.20 on item loading between factors) | All items meet criteria | All items meet criteria | All items meet criteria | All items meet criteria | All items meet criteria |
| (4) | No trivial factors (all factors have at least 3 or more items loading at 0.30 or higher) | Achieved | Achieved | Achieved | Achieved | Achieved |
| (5) | Factors with coefficient alpha > 0.70 | Not achieved | Not achieved | Not achieved | Not achieved | Not achieved |

Notes: Criteria based on Atkins (2014). For “Scree test” of visual representation of eigenvalue plots, see Appendix Figure A1.
